# Two logistic models for the prediction of hypothyroidism in pregnancy

**DOI:** 10.1186/1756-0500-4-205

**Published:** 2011-06-18

**Authors:** Anthony U Mbah, Emmanuel C Ejim, Obinna D Onodugo, Francis O Ezugwu, Matthew I Eze, Peter O Nkwo, Winston C Ugbajah

**Affiliations:** 1 Department of Pharmacology & Therapeutics, College of Medicine, University of Nigeria, Enugu Campus, Enugu, Nigeria; 2 Department of Medicine, College of Medicine, University of Nigeria, Enugu Campus, Enugu, Nigeria; 3 Department of Obstetrics and Gynecology, College of Medicine, Enugu State University of Science and Technology, Enugu, Nigeria; 4 Department of Obstetrics and Gynecology, College of Medicine, University of Nigeria, Enugu Campus, Enugu, Nigeria; 5 Radioimmunoassay Unit, Department of Chemical Pathology, University of Nigeria Teaching Hospital, Enugu, Nigeria

**Keywords:** Pregnancy, Thyroid hypo function, Prediction, Screening, Logistic models

## Abstract

**Background:**

The mounting evidence linking hypothyroidism during pregnancy with poor pregnancy outcome underscores the need for screening and, therefore, a search for more reliable and cheaper screening methods.

**Methods:**

The study was conducted in two phases. The phase one study comprised of healthy women in different stages of pregnancy who attended routine antenatal clinic at St Theresa's Maternity Hospital, Enugu, Nigeria from September 6 to October 18 1994. In this study the variables compared between the hypothyroid and non-hypothyroid pregnant women were maternal age, the number of the pregnancy or gravidity, gestational age, social class, body weight, height, the clinically assessed size of the thyroid gland, serum free thyroxin (FT4) and serum thyrotrophin (TSH). Based on the parameter differences between the two comparison groups of pregnant women two Logistic models, Model I and Model 11, were derived to differentiate the hypothyroid group from their non-hypothyroid counterparts. The two logistic models were then applied in a prospective validation study involving 197 pregnant women seen at presentation in Mother of Christ Specialist Hospital and Maternity, Ogui Road, Enugu from March 2002 to November 2007

**Findings:**

The findings were that 82 (50.3%) of the 163 pregnant women had thyroid gland enlargement while 60 (36.8%) had hypothyroidism as defined by FT4 values below and/or TSH above their laboratory reference ranges. The pregnant subjects with hypothyroidism, compared with their non-hypothyroid counterparts, were characterized by a higher gravidity (p < 0.01), a higher body weight (p < 0.01), a higher goiter prevalence rate (p < 0.01) and a more advanced gestational age (p < 0.0001). A significant, positive correlation was also found between body weight and gestational age (r = 0.5; p < 0.01) At the cut-off point for Model l (fitted with gravidity, thyroid size and gestational age) it had a sensitivity of 100%, a specificity of 72.8% and an overall predictive accuracy of 82.9%; whereas for Model II (fitted with gravidity, thyroid size and body weight) the sensitivity was 100%, the specificity was 59.2% and the overall accuracy of discrimination was 74.8%. In the prospective validation study both models showed a sensitivity of 100% each with specificities of 85.5% for Model I and 76.2% for Model II.

**Conclusion:**

It is concluded that logistic models fitting gravidity, thyroid gland size and gestational age or body weight are useful alternatives in screening for hypothyroidism during pregnancy. There is, however, a need for further independent confirmation of these findings.

## Introduction

An estimated two billion individuals worldwide have insufficient iodine intake, with those in south Asia and sub-Saharan Africa particularly affected [1]. Iodine deficiency has many adverse effects on growth and development. These effects are due to inadequate production of thyroid hormones and are termed iodine-deficiency disorders. Iodine deficiency is the commonest cause of preventable mental impairment worldwide [2]. Iodine deficiency disorders (IDD) also include various degrees of thyroid gland enlargement and thyroid hypofunction. Pregnancy constitutes an increased stress on the thyroid gland, reflecting an increased demand on the maternal thyroid hormone production to meet both fetal and maternal needs. Pregnancy is, therefore, an aggravating factor for IDD [3,4]. There is increasing evidence that even mild degrees of hypothyroidism during pregnancy, if not treated, is associated with poor outcome of the gestation, including fetal neuro-intellectual underdevelopment which manifests as varying degrees of learning disabilities later in life [5-7]. In many developing countries where IDD is still a public health problem the overall negative impact on national development is incalculable [8].

Although there may be little difficulty in recognizing clinically overt hypothyroidism in pregnancy, the same cannot be said of sub-clinical hypothyroidism and hypothyroxinaemia, both types of which have also been associated with fetal neuro-intellectual underdevelopment [6,7]. The latter can only be detected with the expensive and technically sophisticated hormonal assay techniques as are currently available for the assessment of thyroid function. These hormonal assay methods are certainly not cost-effective for use in entire population screenings, especially in IDD-affected countries that may have been already impoverished from the effect of the IDD itself [8,9].

The foregoing has made it more urgent to search for easier and cheaper alternative methods adaptable for population screening of pregnant women for various degrees of hypothyroidism. The interrelationships observed in the initial study between key gestational variables and the chemical status of thyroid function in pregnancy provided a veritable tool for the formulation and testing of the hypothesis that gestation-related clinical variables are useful in the prediction of gestational hypothyroidism.

## Methods

The Ethics Committee of University of Nigeria Teaching Hospital (UNTH), Enugu, approved this study prior to its take-off. All the pregnant women who attended routine antenatal clinic at Saint Theresa's Maternity Hospital in Enugu, Nigeria from September 6 to October 18, 1994 and who gave informed consent were screened for eligibility to participate in the initial study. This was done using an entrance checklist designed to exclude persons with evident concomitant acute or chronic illnesses and those using drugs known to independently alter the thyroid function or affect the assay results [10,11]. Out of the 222 pregnant women seen during the study period, 163 satisfied the inclusion criteria and so were used for the current analyses. The study hospital runs a busy general obstetrics practice and so was chosen partly to avoid referral bias. Each subject was studied once during which the age, the gestational age, the number of the pregnancy or gravidity, the social class (scored 1-5 for the highest class-the lowest class). The scores were obtained by averaging each patient's two other scores; one for education and the other for occupation using the system developed by Oyedeji and described in detail elsewhere [12]. The height and the body weight were documented. The gestational age of the subjects was calculated from the last menstrual period (L.M.P.) and/or determined by measurement of the symphysio-fundal height where the L.M.P was indeterminate, as is done conventionally. The size of the thyroid gland was assessed clinically and classified by size into 0, IA, IB, 2 and 3 using World Health Organization's (W.H.O's) recommended criteria [1]. One of the authors (AUM) carried out all the thyroid examinations and did the goiter classifications. A single 5 ml venous blood sample was also taken from each of the participants for serum free thyroxine and serum thyrotrophin assays. A prior decision was to define hypothyroidism as FT4 below the laboratory reference range of 9.5-23.6 pmo1/L and/or TSH above the laboratory reference range of 0.5-6.0 mU/L. All the assays were run in duplicates. FT4 was determined using commercial kits that employ magnetic solid phase separation and enzyme immunoassay methods (Serrano Diagnostics, Coinsins, Switzerland). TSH assays were carried out using the two-step immunoradiometric assay (IRMA) with bulk reagents from NETRIA of London, England. The lower detection limits for FT4 and TSH were 0.06 pmoI/L and 0.01 mU/L respectively, while the intra-assay and inter¬-assay coefficients of variation were both ≤ 6.4%.

### Prospective validation

In a limited prospective validation study all the pregnant volunteers who gave informed consent participated in the study. They were recruited at booking from Mother of Christ Specialist Hospital and Maternity, Ogui Road, Enugu from March 2002 to November 2007. The gestational age, gravidity, body weight, thyroid size, TSH and FT4 values were determined using the same methods and techniques as already described in the initial study. The data entry and analysis were done at the end of the study, as was decided upon prior to the onset of the validation study.

### Statistical analysis

The statistical analysis was performed using the Statistical Package for the Social Sciences version 13 (SPSS-13) [13] run on a compatible personal computer. The data collected in the initial study were all examined for distributional patterns, at first visually using quantal-¬quantal plots and then confirmed at p > 0.05 using the Shapiro-Wilk Normality test. Subsequently, the data were compared between the hypothyroid and the non-¬hypothyroid pregnant women employing parametric t-tests and non-parametric Mann-Whitney U tests as appropriate for normally and non-normally distributed data respectively. The Chi-square test was used to analyze binary data. Correlation coefficients and their levels of statistical significance were determined using simple linear regressions and the Spearman's non-parametric assessment of co-linearity. The level accepted as statistically significant was if p < 0.05. The use of discriminant analyses in providing solutions to problems involving classification into groups has been extensively reviewed [14,15]. Multivariate linear models, although very simple, were considered inappropriate in this case because of the variegated nature of our data [14]; continuous, discrete and binary data were fitted together in one model.

Multivariate logistic analyses were performed in which the variables entered were those that differed significantly between the two comparison groups. For the purpose of the logistic regression analyses the thyroid size was recorded as follows: (i) gland not visible when the neck is in the normal position (grades 0, IA and IB according to the World Health Organization's classification) = 1, (ii) gland visible when the neck is in the normal position (grades 2 and 3 according to the World Health Organization's classification) = 2. This modification was done solely for computational convenience.

The fitted models belong to the binomial family of the Generalized Linear Models [15] with the Logit link function. These models are based on the general assumption that ln[p(H)/(1-p(H)] = α+b_1_x_1_+b_2_x_2_+b_3_x_3_...+b_n_x_n_; where ln is the Naperian logarithm, p(H) is the predicted probability for chemical hypothyroidism, α+b_1_x_1 _+b_2_x_2 _+b_3_x_3_+b_n_x_n _is the linear predictor of the logistic regression function in which α is the intercept, b_1, _b_2, _b_3_...b_n _are the coefficients and x_1, _x_2, _x_3..._x_n _are the predictor variables. Transforming the same basic equation gives p(H) = e^bx^*1*(1+e^bx^); where bx is the linear predictor α+b_1_x_1_+b_2_x_2_+b_3_x_3_...+b_n_x_n_. and e is the base of the Naperian logarithm. Gestational age and body weight were significantly correlated and, as a rule, both should not be entered in the same model [15]. Two alternative models (Model I and Model II) were, therefore, derived. The variables entered in Model I were gravidity, goitre score and gestational age; whereas in Model II the variables were gravidity, goitre score and body weight. The variables were fitted using the Maximum Likelihood method [15]. Receiver Operating Characteristics (ROC) data generated for each of the two models (performances at each of the predicted probability levels for the patients with hypothyroidism) were used to construct ROC curves whose slopes and area under the curves (AUCs) were further compared for statistical significance.

### Analysis of the prospective validation data

Using the MLAB Mathematical and Statistical Modeling package [15] the p(H) for each subject was calculated automatically after imputing into the computer Gravidity, Goiter score and Gestational age for Model I; and Gravidity, Goiter score and Body weight for Model II. Subjects with p(H) values compatible with hypothyroidism were then identified using the already established cut-off values for each of the two models. The chemical indices of hypothyroidism found among the subjects were then compared against the individual's predicted probability of hypothyroidism, p(H). The accuracy of the predictions was used in assessing the performance of each of the two logistic models. The criteria used to define hypothyroidism in the validation study were the same as those used in the initial study.

## Results

Results are stated as Mean ± Standard error of the mean (SEM) for normally distributed data; Minimum-Maximum range (median) for non-normal as well as categorical data; and percent (%) for binary data. In the initial study the 163 subjects were aged 29.9 ± 0.8 years (mean ± SEM), their mean gestational age was 28.4 ± 0.6 weeks, their range of gravidity was l-8 (median = 3), their range of scores for social class was 1-5 (median = 4), their height ranged 1.40 m-¬1.90 m (median = 1.60 m) while their mean body weight was 75.2 ± I.4 kg. The overall goiter prevalence rate found among the patients was 50.3%. The observed range of FT4 was 5.4-26.1 pmol/L (median = 13.8 pmol/L) while that for TSH was 0.0-10.6 mU/L (median = 4.9 mU/L). The laboratory reference ranges for these parameters were 0.5-6.0 mU/L for the TSH and 9.5-23.6 pmo1/L for the FT4. Out of all the participants in the initial study 60 (36.8%) met the diagnostic criteria for hypothyroidism. Out of this number 47 or 78.3% had sub-clinical hypothyroidism (high TSH with normal FT4), 12 or 20% had hypothyroxinaemia (normal TSH with low FT4) while only one patient had overt hypothyroidism (high TSH with low FT4). In (table 1), the demographic data and total goiter prevalence rates were compared between those with hypothyroidism and their non¬-hypothyroid counterparts. It was found that the subjects with hypothyroidism were significantly older (p < 0.01), had a higher gravidity (p < 0.001), weighed more (p < 0.001), were more goitrous (p < 0.01) and were at a more advanced gestational age (p < 0.001). The relationship between age and gravidity as well as between gestational age and body weight was further investigated using Spearman's and simple linear regression correlation coefficients respectively. Whereas the coefficient of correlation between gestational age and body weight was statistically significant (r = 0.5; p < 0.01) that between age and gravidity was not (r = 0.06; P > 0.05).

**Table 1:** A comparison of the demographic and goitre data between the hypothyroid and non-hypothyroid pregnant women in the initial study.

Parameter	Subjects Without Hypothyroidism (N = 103)	Subjects With Hypothyroidism (N = 60)	P-Value
**Age (Yrs)**	22.9 ± 0.6	25.4 ± 0.7	< 0.01^#^*
**Gest. Age (Wks)**	23.8 (4.3-33.1)	29.6(16.8-40.1)	< 0.001^+^*
**Social Class (Scores)**	4(1-5)	4(1-5)	> 0.05^+ns^
**Gravidity**	3(1-8)	2(1-6)	< 0.01^+^*
**Weight (Kg)**	63.5(51.3-93.7)	71.0(48.9-102.7)	< 0.01^+^*
**Height (m)**	1.6 ± 0.02	1.6 ± 0.03	> 0.05^#ns^
**Goiter Frequency (%)**	43(71.7%)	39(37.9%)	< 0.01^$^*

N = Number of patients

* = Statistically significant at p < 0.05.

ns = Not statistically significant at p < 0.05

^# ^= Analysis by two-tailed, unpaired Student t-test.

^+ ^= Analysis by Mann-Whitney U test

^$ ^= Analysis by Chi-square test

Yrs = Years

Wks = Weeks

### The logistic regression results

**Figure 1:**
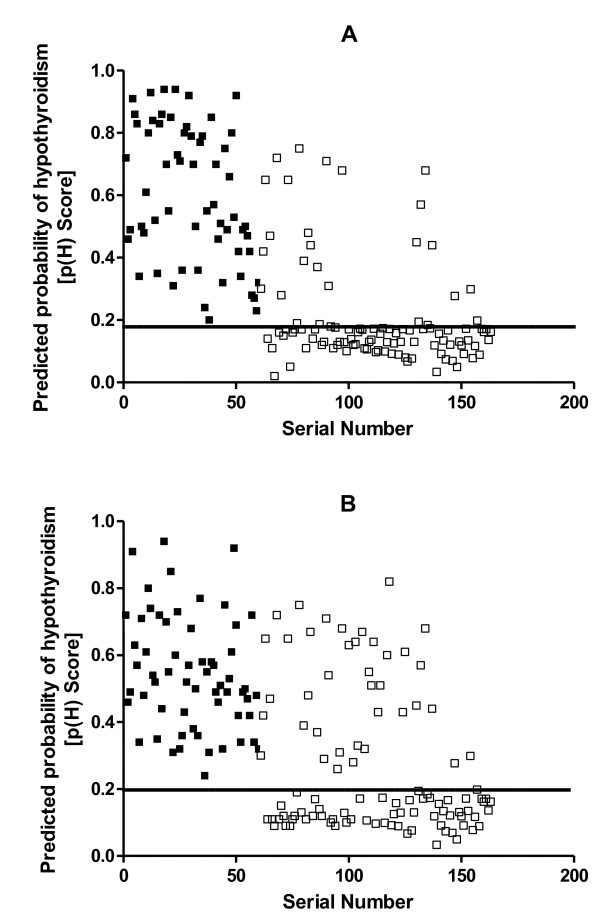
**The predicted probability scores for hypothyroidism, p(H), in the initial study**. The plots show the predicted probability scores plotted against the serial number of each patient for those with hypothyroidism (■) and those without hypothyroidism (□), using model I (A) and using model II (B). The lines parallel to the x-axes show the respective cut-off points for Model I (0.18) and for Model II (0.20). For purposes of clarity, the patients were numbered after sorting them in the descending order of their TSH values.

**Figure 2:**
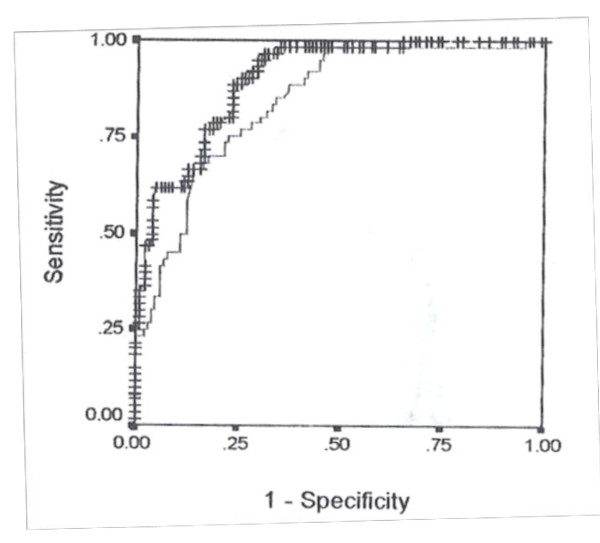
**The receiver operating characteristics (ROC) curves for models I and II in the initial study**. The receiver operating characteristics (ROC) curve for model I is indicated by crosses (++++) while that for model II is indicated by broken lines (----). The ROC data used was generated by determining the sensitivities and specificities of each model at different cut-off points of the predicted probability scores of the patients with hypothyroidism.

In model I the variables entered were age, gravidity, goiter score and gestational age; while in model II they were age, gravidity, goiter score and body weight. The final models (I and II) did not include age since the removal of this variable did not affect the deviance of either model significantly (χ*^2 ^*= 1.759; df = 1; P > 0.05 for model I and χ*^2 ^*= 2.550; df = 1; p > 0.05 for model II). The predicted probability of hypothyroidism, p(H), was determined for each of the pregnant women in conformity with the general logistic function: p(H) = e^bx^/(1 + e^bx^). Tables 2 and 3 show the coefficients of the logistic regressions, with their standard errors and levels of statistical significance, for Models I and II respectively. When the regression coefficients for model I were substituted in the linear predictor its value was determined by: bx = -2.1001 + 0.0286 × GRAVIDITY + 0.0717 × GOITRE SCORE + 0.0413 × GEST.AGE (in weeks.). For model II also bx = -3.6496 + 0.2661 × GRAVIDITY + 0.4063 × GOITRE SCORE + 0.0124 × WEIGHT (in kilogrammes). Figures 1 and 2 show respectively plots of predicted probabilities, p(H), against the serial number of each patient for Models I and II. There were evidently more false-positive scores with Model II than with Model I. The Receiver-Operator Characteristics (ROC) curves for the two models are shown superimposed in Figure 2. An analysis of the two Receiver-Operator Characteristics (ROC) curves showed a left-shift position for Model I, an indication of its better performance compared with Model II. Although the ROC curve for Model I showed a steeper slope compared with that of model II (p < 0.001), there was, however, an insignificant difference in the area under the curves (AUCs) for both models (95% Confidence interval = -0.029 to 0.457, p > 0.05). The optimal cut-off point of the probability for model I which gave 100% sensitivity was 0.18. At that point its specificity and overall accuracy of discrimination were 74.8% and 82.9% respectively. For model II the optimal specificity achieved at 100% sensitivity was 64.1% and this occurred at the cut-off point of 0.20 where it achieved an overall discrimination accuracy of 74.8%.

**Table 2:** The coefficients of the logistic regressions for model I

Coefficients	Values	Standard Errors	P-Values
**Gravidity**	0.0286	0.0104	< 0.05*
**Goiter Score (1-2)**	0.0717	0.0118	< 0.05*
**Gest. Age (Wks)**	0.0413	0.0027	< 0.001*
**Intercept**	-2.2001	0.0606	< 0.001*

* = Statistically significant at p < 0.05

Goiter Scores: 1 = Goiter palpable but invisible with neck in the normal position; 2 = Goiter visible with neck in the normal position

Wks = Weeks

**Table 3:** The coefficients of the logistic regressions for model II

Coefficients	Values	Standard Errors	P-Values
**Gravidity**	0.2661	0.1661	< 0.05*
**Goiter Score (1-2)**	0.4063	0.1064	< 0.05*
**Weight (Kg)**	0.0124	0.0192	< 0.001*
**Intercept**	-3.6496	1.1032	< 0.001*

* = Statistically significant at p < 0.05

Goiter Scores: 1 = Goiter palpable but invisible with neck in the normal position; 2 = Goiter visible with neck in the normal position

Wks = Weeks

### The results of the prospective validation study

A total of 197 pregnant women participated in this prospective validation study. Their mean age was 23.8 ± 1.3 years, their mean gestational age was 22.5 ± 0.9 weeks and their mean body weight was 53.7 ± 1.7 kg. Their gravidity in minimum-maximum range(Median) was 1-5(1.87). Goiter was prevalent in 18 (9.13%). The largest goiter size encountered was stage IB (W.H.O. Classification). The TSH was 1.8 mU/L-6.9 mU/L(4.0 mU/L) while the FT4 was 7.9 pmol/L-28.2 pmol/L(16.85 pmol/L). Out of the 197 pregnant women in this study 12 (6.09%) actually had hypothyroidism as defined by the afore-mentioned criteria. Table 4 shows a comparison of the most relevant demographic and thyroid function data between the subjects in the initial study and those in the validation study. With reference to these parameters the two groups were found to differ significantly, with those in the initial study being much older (p < 0.001), having more advanced gestational age (p < 0.0001) and weighing much more (p < 0.0001). The subjects in the initial study were also found to have had more pregnancies (p < 0.001) and had a greater frequency and size of thyroid gland enlargement or goiter (p < 0.0001). Interestingly, the subjects in the initial study were also characterized by a higher TSH and a lower FT4 when compared with those in the validation study (p < 0.001 and p < 0.0001 respectively).

**Table 4:** A comparison of the demographic, goiter and thyroid function data between the pregnant women in the initial study and those in the validation study.

PARAMETER (Unit)	INITIAL STUDY VALUE: N = 163	VALIDATION STUDY VALUE: N = 197	P-Value
**Age (Yrs)**	29.9 ± 0.8	23.8 ± 1.3	< 0.001^#^*
**Gestational Age (Wks)**	28.4 ± 0.6	22.5 ± 0.9	< 0.0001^#^*
**Gravidity**	3.0(1-8)	1.9(1-5)	< 0.001^+^*
**Goiter Prevalence Rate (%)**	50.3	9.1	< 0.0001^$^*
**Body Weight (Kg)**	75.2 ± 1.4	53.7 ± 1.7	< 0.0001^#^*
**TSH (mU/L)**	4.9(00-10.6)	4.0(1.8-6.9)	< 0.001^+^*
**FT4 (pmol/L)**	13.8(5.4-26.1)	16.9(7.9-28.2)	< 0.0001^+^*

N = Number of subjects

* = Statistically significant at p < 0.05

^# ^= Analysis by two-tailed, unpaired Student t-test

^+ ^= Analysis by Mann-Whitney U test

^$ ^= Analysis by Chi-square test

Yrs = Years

Wks = Weeks

**Figure 3:**
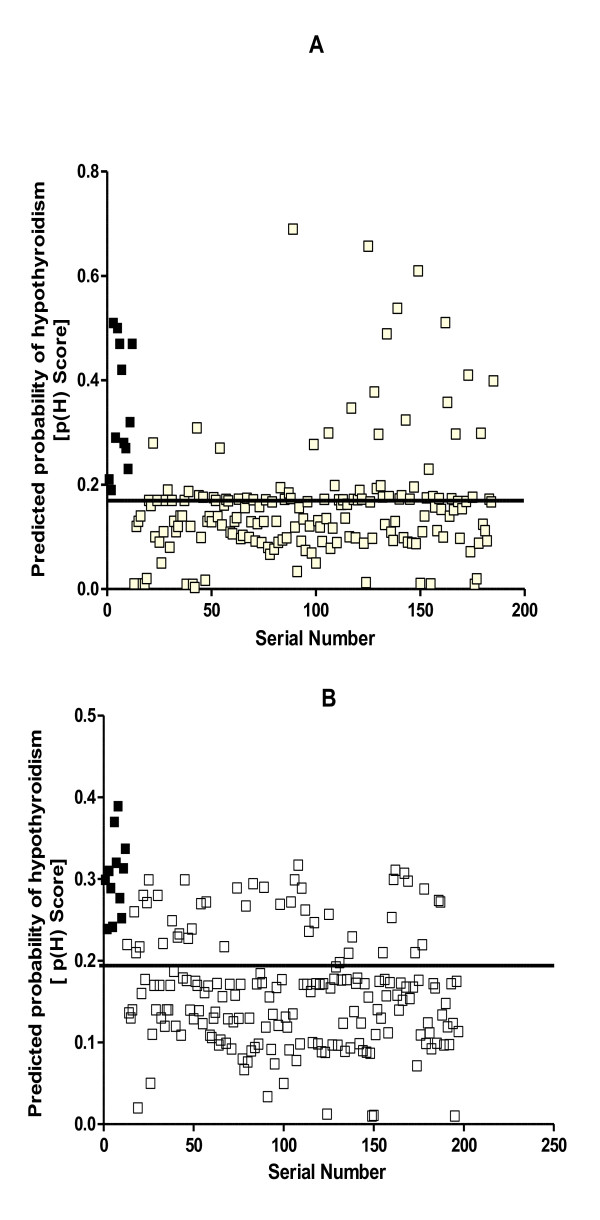
**The predicted probability scores for hypothyroidism, p(H), in the validation study**. The plots show the predicted probability scores plotted against the serial number of each patient for those with hypothyroidism (■) and those without hypothyroidism (□), using model I (A) and using model II (B). The lines parallel to the x-axes show the respective cut-off points for Model I (0.18) and for Model II (0.20). For purposes of clarity, the patients were numbered after sorting them in the descending order of their TSH values.

Figures 3A and 3B display graphically the performances of Model I and Model II respectively. It was found that both models correctly identified all those that had hypothyroidism, thereby giving a sensitivity of 100% for each model and specificities of 85.5% and 76.2% for Models I and II respectively.

## Discussion

Our initial study showed a 36.8% prevalence of hypothyroidism among the pregnant women studied. This prevalence level of hypothyroidism is not uncommon in an environment like Nigeria where iodine deficiency disorders (IDD) are still of public health concern [1-4,16]. In areas of Nigeria with IDD problem the prevalence rates of goiter reported during pregnancy range from 46.8% to 92.7% [16-18]. The goiter prevalence rate of 50.4% found in this study is, therefore, within the range reported in the previous studies. The genesis of endemic goiter in the Eastern part of Nigeria has been traced to a number of staple foods common in this area, in addition to the age-long method of preserving salt over the fire place, thereby depleting the iodine content [19-21]. Although iodine deficiency causes hypothyroidism generally it is more so during pregnancy when the requirement for thyroid hormones is increased with a parallel decrease in the maternal iodine pool [22]. The latter is thought to be due to the gestational decrease in renal threshold for iodine that favours enhanced urinary iodine loss. Our results are, therefore, in support of previous reports that hypothyroidism is a common finding during pregnancy in areas with sub-optimal iodine intake [2-4,23-26]. One of the findings of this study is that the probability of hypothyroidism during pregnancy has a direct relationship to the gestational age. This is consistent with other previous reports indicating a similar relationship [3,25].

Hypothyroidism during pregnancy, even if it is of a mild degree, is associated with increased risk of fetal abnormalities [4-7]. However, it is uncertain whether the high prevalence observed in our initial study also connotes a high prevalence of fetal abnormalities. This is because fetal screening was not part of the study design. Screening for and treating hypothyroidism during the neonatal period is routinely done in many developed countries today [26]. However, routine screening of pregnant women for hypothyroidism has not yet become established practice, despite the numerous experimental data indicating that the adverse fetal consequences of maternal hypothyroidism in pregnancy are preventable by maternal supplementation with iodine and/or L-thyroxin [27,28]. This is presumptive of a need for routine screening for occult hypothyroidism among pregnant women and for replacement therapy in affected cases [8].

In addition to the stress occasioned by the increased thyroxin demand on the maternal thyroid gland, pregnancy is also associated with a marked increase in serum thyroxin-binding globulin (TBG) levels in late pregnancy as well as an increase in the thyrotrophic effect of human chorionic gonadotrophin (hCG), especially in early pregnancy [29]. The gestational increase in maternal thyroxin demand, coupled with other gestational metabolic changes, results in profound, compensatory alterations in the size and function of the thyroid gland [23,29,30]. In areas with sub-optimal iodine intake the observed trend is an increase in maternal basal serum TSH level and/or a decrease in serum FT4; a trend that has been shown to increase in intensity with advancing gestational age up to the time of delivery [26]. The relative trends in TSH and FT4 values found in the initial and the validation studies are in keeping with these observations. High TSH and low FT4 have been shown to be independent risk factors for the outcome of pregnancy [31,32]. Besides, the compensatory morphological changes observed in the maternal thyroid gland have been shown to persist far into the post-partum period; leading to the widely accepted conclusion that the thyroidal effect of subsequent pregnancies is a cumulative aggravation, irrespective of whether they end up in term delivery or in abortion [33,35]. The latter may be part of the explanations for the direct relationship found in the current study between the probability of gestational hypothyroidism and goiter as well as the gravidity of the women. This fact also informed our preference of gravidity to parity as a parameter of interest in the current investigation.

Although clinically overt hypothyroidism may be easily recognized in pregnant women on the basis of symptoms and signs, milder degrees of hypothyroidism may go unnoticed because affected pregnant women clinically appear healthy. The only way to detect these is by performing the chemical tests of thyroid function. Chemical tests of thyroid function, if used for the routine screening of all pregnant women living in high-risk areas for IDD, would certainly be too costly an enterprise and, therefore, unaffordable. A search for cheaper and simpler, yet reliable methods of preliminary screening for hypothyroidism in pregnancy has, therefore, become imperative. It is this consideration that has necessitated the current study, one of the objectives of which is to examine the possible use of some clinical parameters in the prediction of clinically occult hypothyroidism among pregnant women. Although such a finding, even if confirmed, is unlikely to replace the biochemical tests of thyroid function it may go a long way as an initial screening tool in order to save cost.

The statistically significant differences in gestational age, gravidity, goiter rates and body weight as found between the hypothyroid and non-hypothyroid pregnant women in the initial study offered a good opportunity for the formulation and testing of the afore-stated study hypothesis. The relationship of these same variables to the gestational status of thyroid function has been described in previous, non-Nigerian studies [3,4]. However, to the best of our knowledge, there has not been any previous attempt to fit discriminant models to these clinical data in an attempt to predict the presence or absence of hypothyroidism during pregnancy. The two Models developed, Model I (goiter score, gravidity and gestational age fitted) and Model II (goiter score, gravidity and body weight fitted) both theoretically showed good performance as evidenced by their high sensitivities, good specificities and reasonable overall discrimination abilities. If confirmed this could mean a kindling of hope for poor countries at risk for IDD, where facilities for all-inclusive biochemical screening for hypothyroidism among pregnant women is either unavailable or unaffordable.

The earliest time that it becomes practicable to screen pregnant women medically is during the time of booking. It is this understanding that informed the focusing of the validation study on subjects who came for booking. The mean gestational age of the women used in the current validation study is in agreement with what has been reported previously in the literature among pregnant Nigerian women at booking [36-39]. The younger gestational age of the subjects in the validation study, compared with those in the initial study, may also partly explain the significant differences found between the two groups with respect to the other clinical parameter values.

The subjects in the prospective validation study, when compared with those in the initial study, were also found to have significantly lower TSH and higher FT4 indicative of relative hyperthyroidism. More recently, longitudinal studies among pregnant women have widened our understanding of some of the mechanisms of gestational thyroid function regulation. The first half of pregnancy is associated with high levels of human chorionic gonadotrophin (hCG) [40-42] while the second half is characterized by high levels of thyroxin-binding globulin (TBG) [43,44] as well as a reduction in the renal threshold for iodine which results in its increased urinary loss [45,46]. That hCG has a thyrotrophic effect is a well documented fact [29,40] and this may be part of the explanations for the relative hyperthyroidism in the validation study compared with what was found in the initial study. It is possible that differences in the concentrations of hCG and TBG between the two groups may help in explaining the parameter differences observed. It is interesting; however, that despite these differences Model I and Model II both fitted the validation data in which both exhibited 100% sensitivity each. It is uncertain whether or not, and if so to what extent, this finding may have been influenced by the low prevalence rate of hypothyroidism in the validation study.

In conclusion therefore, these results are supportive of the study hypothesis that models based on clinically obtainable information can be predictive of maternal hypothyroidism in pregnancy. If confirmed, such models could be cost-saving and, therefore, veritable epidemiologic tools for maternal thyroid function screening during pregnancy; more especially in settings with limited iodine intake. Although the evidence provided in the current study appears compelling, it is still far from being conclusive. One of the considered achievements of the current exposition is that a new direction of focus may have been chatted out as grounds for further research.

## Competing interests

The authors declare that they have no competing interests.

## Authors' contributions

MUA conceived of the study, and participated in its design and coordination and helped to draft the manuscript. The thyroid gland examination, the classification of goiters and their documentation were done by him exclusively. He also made the greatest intellectual input into this paper. This was because he had the vastest field experience, having done a lot of work in the endemic goiter areas of South-east Nigeria.

ECE made substantial contributions towards the conception, design, data acquisition, data interpretation and in the preparation of the manuscript.

ODO made substantial contributions towards the conception, design, data acquisition, data interpretation and in the preparation of the manuscript.

EOF made substantial contributions towards the conception, design, data acquisition, data interpretation and in the preparation of the manuscript.

EIM made substantial contributions towards the conception, design, data acquisition, data interpretation and in the preparation of the manuscript.

NOP contributed towards the data acquisition and has been involved in the drafting of the manuscript and revising it critically for important intellectual content.

UCW made substantial contributions towards the conception, design, data acquisition, data interpretation and in the preparation of the manuscript.

All the authors read and approved the final manuscript.
